# Thrombin Generation in Acute and Chronic Liver Disease in Children

**DOI:** 10.3390/diagnostics16091328

**Published:** 2026-04-28

**Authors:** Giovina Di Felice, Anna Lisa Montemari, Andrea Pietrobattista, Luca Della Volpe, Antonella Mosca, Daniela Liccardo, Simona Pezzi, Chiara Giorni, Matteo Luciani, Danilo Alunni Fegatelli, Annarita Vestri, Ottavia Porzio

**Affiliations:** 1 Clinical Laboratory Unit, Bambino Gesù Children’s Hospital, IRCCS, 00165 Rome, Italy; annalisa.montemari@opbg.net (A.L.M.); simona.pezzi@opbg.net (S.P.); ottavia.porzio@opbg.net (O.P.); 2 Division of Metabolic Diseases and Hepatology, Bambino Gesù Children’s Hospital, IRCCS, Full Member of the European Reference Network Rare Liver, 00165 Rome, Italy; andrea.pietrobattista@opbg.net (A.P.); luca.dellavolpe@opbg.net (L.D.V.); antonella.mosca@opbg.net (A.M.); daniela.liccardo@opbg.net (D.L.); 3 Pediatric Cardiac Intensive Care Unit, Bambino Gesù Children’s Hospital, 00165 Rome, Italy; chiara.giorni@opbg.net; 4 Pediatric Hematology and Oncology Unit, Bambino Gesù Children’s Hospital, IRCCS, 00165 Rome, Italy; matteo.luciani@opbg.net; 5 Department of Life Sciences, Health and Health Professions, Link Campus University, 00165 Rome, Italy; danilo.alunnifegatelli@gmail.com; 6 Department of Public Health and Infectious Diseases, Sapienza University, Policlinico Umberto I, 00161 Rome, Italy; annarita.vestri@uniroma1.it; 7 Department of Experimental Medicine, University of Rome Tor Vergata, 00133 Rome, Italy

**Keywords:** pediatrics, coagulation, thrombin generation, chronic liver disease, acute liver diseases

## Abstract

**Background:** Pediatric liver disease is frequently associated with abnormal conventional coagulation tests; however, prothrombin time expressed as international normalized ratio (PT-INR) incompletely reflect global hemostatic balance. Thrombin generation assay (TGA) provide an integrated assessment of coagulation and may offer complementary information in children with acute liver failure (ALF) and chronic liver disease (CLD). **Methods:** We enrolled 61 pediatric patients with liver disease (50 CLD, 8 ALF, 3 extrahepatic portal vein obstruction EHPVO) and 51 healthy controls. Platelet-poor plasma was prepared according to international recommendations. Thrombin generation was measured using ST Genesia (STG) with normalization to reference plasma. Group comparisons were performed using non-parametric tests; correlations between PT-INR and thrombin generation parameters were assessed, and principal component analysis (PCA) was used to explore the variance structure of thrombin generation indices and conventional coagulation variables. **Results:** PT-INR was significantly higher in patients than controls, particularly in ALF. Bleeding events were uncommon. Compared with controls, patients showed reduced levels of fibrinogen and multiple procoagulant/anticoagulant factors (including antithrombin and protein C), with increased factor VIII. Among thrombin generation parameters, the endogenous thrombin potential (ETP) ratio differed significantly across groups (*p* = 0.001), while correlations between PT-INR and thrombin generation parameters were weak or absent, no significant associations were observed even at higher Pediatric/Model for End-Stage Liver Disease scores. PCA separated thrombin generation indices from PT-INR and conventional coagulation factors, suggesting complementary information. **Conclusions:** In pediatric liver disease, PT-INR does not reliably reflect global coagulation capacity. Thrombin generation testing provides additional, integrative information on hemostasis and may improve laboratory assessment beyond conventional tests.

## 1. Introduction

Thrombin generation plays a central role in hemostasis, integrating procoagulant and anticoagulant mechanisms and influencing both clot formation and fibrinolysis [1]. Because the liver synthesizes most coagulation factors and natural anticoagulants, hepatic dysfunction profoundly affects coagulation pathways. This is observed in both acute liver failure (ALF) [2] and chronic liver disease (CLD) [3] in adults and children, although the pediatric population shows age-dependent physiological differences in baseline hemostasis and in response to liver injury.

Liver disease may be associated with either bleeding or thrombosis, reflecting a fragile and dynamic “rebalanced hemostasis” rather than a uniform hypocoagulable state [4]. Thrombocytopenia and platelet dysfunction are common and may further complicate the clinical picture [5]. However, PT-INR primarily reflects reductions in procoagulant factors and does not incorporate changes in anticoagulant proteins, platelet contribution, and fibrinolysis, limiting their ability to predict bleeding risk in liver disease.

Alterations in fibrinolysis also contribute to the hemostatic phenotype of ALF. Reduced plasminogen levels and increased plasminogen activator inhibitor-1 (PAI-1) may lead to a hypofibrinolytic state and potentially increase thrombotic risk [6]. In this setting, a combination of thrombocytopenia partially counterbalanced by elevated von Willebrand factor and factor VIII, reduced ADAMTS-13 activity, hypofibrinolysis, and procoagulant microparticles may collectively contribute to thrombotic complications and worsen outcomes. In pediatric ALF, reduced factor V and factor VII levels have been associated with poor outcomes and may provide prognostic information [7].

In cirrhosis and end stage liver disease (ESLD), progressive fibrosis and hepatocellular dysfunction lead to long-term disturbances in both procoagulant and anticoagulant pathways. Thrombin generation may decrease as hepatic synthetic capacity deteriorates [8]; nevertheless, advanced disease can be associated with a relatively hypercoagulable profile driven by increased factor VIII and reduced protein C and antithrombin [9]. This imbalance may contribute to venous thromboembolism and portal vein thrombosis, particularly in decompensated cirrhosis [10].

In pediatrics, thrombosis is generally less frequent than in adults [11], and bleeding events remain a major clinical concern. Paradoxically, despite prolonged PT-INR, major bleeding is often less common than expected [12]. In routine clinical practice, prolonged PT-INR is often interpreted as a marker of bleeding risk and may influence decisions such as plasma transfusion or the postponement of invasive procedures. However, PT-INR does not reflect the balance between procoagulant and anticoagulant pathways and has been shown to be a poor predictor of bleeding in liver disease. Therefore, reliance on PT-INR alone may lead to inappropriate clinical management and highlights the need for more comprehensive assays of hemostasis.

Kawada et al. describe that, in pediatric liver disease, PT-INR is more a marker of impaired hepatic synthetic function than a reliable predictor of bleeding risk. Consequently, the use of global hemostatic assays such as thromboelastography (TEG/ROTEM) and thrombin generation assay (TGA) provides additional insight into coagulation processes, rather than relying on INR alone [13].

In this context, thrombin generation assays may provide a global assessment of coagulation potential and support more informed clinical decision-making.

The aim of this study was to characterize coagulation profiles in pediatric patients with acute and chronic liver disease of varying severity, with a particular focus on thrombin generation parameters and their relationship with PT-INR.

## 2. Materials and Methods

### 2.1. Subjects

A total of 112 subjects aged 1–18 years were enrolled, including 61 patients with liver disease and 51 healthy controls. Patients scheduled in division of metabolic diseases and hepatology pediatric surgery, admitted to Bambino Gesù Children’s Hospital between June 2023 and June 2024. Disease severity was evaluated using the Pediatric End-Stage Liver Disease (PELD) and Model for End-Stage Liver Disease (MELD) scores [14] applying a predefined cut-off of 10 to classify patients into Group A (≤10) and Group B (>10). No patients received vitamin K supplementation 5 days prior to sample collection.

Controls were recruited among children attending Bambino Gesù Children’s Hospital for routinary blood testing/check-ups, who met the eligibility criteria and had no evidence or history of liver disease or other relevant comorbidities.

Inclusion criteria:

- Diagnosis of chronic liver disease (any severity) or acute liver failure;
- Age between 1 and 18 years;
- Male and female sex;
- Caucasian ethnicity;
- Written informed consent from a legally authorized representative (parents/legal guardian), in accordance with national regulations.

Exclusion criteria:

- Known congenital coagulation disorders;
- Ongoing anticoagulant therapy;
- Acute or chronic infection within 30 days prior to enrollment.

This study was approved by the Institutional Ethics Committee of Bambino Gesù Children’s Hospital (certificate no. 3152_OPBG_2023) and conducted in accordance with the Declaration of Helsinki.

### 2.2. Sample Collection and Handling

Venous blood was obtained by peripheral venipuncture from the antecubital vein using a 23-gauge needle, applying a tourniquet for no longer than 1 min to avoid venous stasis. Samples were collected between 7:00 and 9:00 a.m. for all patients. Blood was drawn into pediatric Vacutainer tubes (Greiner Bio-One, Kremsmünster, Austria), which have the same external dimensions as adult tubes but allow the collection of a limited blood volume (1.8 mL). The tubes contained 3.2% sodium citrate as anticoagulant at a ratio of 1:9 (*v*/*v*, sodium citrate:blood), in accordance with international recommendations [15,16]. The tubes were gently inverted 5–6 times immediately after collection to ensure proper mixing of blood with the anticoagulant. Within 1 h of collection, platelet-poor plasma (PPP) was prepared by centrifuging the blood at 2000× *g* for 15 min at 20 °C. Immediately thereafter, the supernatant was recovered and centrifuged again under the same conditions. PPP was defined as plasma containing <10,000 platelets/µL. PPP was aliquoted (0.5–1.0 mL) into labeled polypropylene tubes and stored at −80 °C until analysis. Plasma samples were processed within 6 months of collection and thawed in a 37 °C water bath for 1–5 min. After thawing, samples were mixed and analyzed within 2 h [15,16]. All collected samples were suitable for analysis. No patients presented with elevated hematocrit levels.

### 2.3. Laboratory Assays—Thrombin Generation

Thrombin generation (TGA) was measured using the ST GenesIIa platform (Diagnostica Stago, Asnières-sur-Seine, France).

The first step was to run a calibration curve. This calibration curve was performed with the STG-Cal Fluo kit, which contained STG- Thrombical, STGFluoStart and STG Fluoset. The STG ThrombiCal contained the calibrator (Thrombical activity 596 nM). The STG-FluoStart contained the substrate and calcium; the STG FluoSet contained a fluorophore product that was used for calibration of the sample. Once the calibration was performed successfully, the reference plasma and quality controls could be measured. This reference plasma came with specific assigned ranges provided on a barcoded flyer and helped to normalize results. Normalized results for each sample were calculated by applying the formula [patient sample result/reference plasma result x activity assigned for the particular lot and parameter of this reference plasma]. This normalization calculation was done automatically by the software of the analyzer. Patient and control PPP samples were tested using the STG-BleedScreen kit (Diagnostica Stago, Asnières-sur-Seine, France). Fluorescence was recorded every 15 s (excitation 377 nm; emission 450 nm). Thrombin generation assays are global hemostasis tests that describe the kinetics and magnitude of thrombin formation over time. The following parameters were analyzed: lag time, time to peak, and start tail (expressed as ratios), as well as peak height, endogenous thrombin potential (ETP), and velocity index (expressed as percentages), according to the assay output.

### 2.4. Statistical Analysis

Statistical analyses were performed using R (version 4.5.0). Descriptive statistics were computed for all variables. Continuous variables were summarized as mean (standard deviation, SD) and median (interquartile range, IQR), whereas categorical variables were reported as counts (percentages).

Boxplots and violin plots were used to visualize the distribution of thrombin generation parameters and other continuous variables across diagnostic groups. Comparisons among groups were performed using the Kruskal–Wallis test. Categorical variables were compared using the chi-square test. Correlations between thrombin generation parameters and clinical/laboratory variables, including INR, were assessed using Spearman rank correlation coefficient. Principal component analysis (PCA) was conducted to explore the underlying structure of thrombin generation parameters, coagulation factors, and INR. Given the exploratory nature of this study, no formal correction for multiple comparisons was applied; therefore, *p*-values should be interpreted descriptively and with caution. Despite this, the results showed a coherent pattern across analyses, supporting the interpretation that thrombin generation parameters provide information complementary to INR and conventional coagulation measures.

## 3. Results

Overall, 112 subjects were enrolled, including 61 patients with liver disease (30 females, 31 males) and 51 healthy controls (29 females, 22 males). Among patients, 50 children were diagnosed with chronic liver disease (CLD), 8 with acute liver failure (ALF), and 3 with extrahepatic portal vein obstruction (EHPVO). Mean age was 11.1 ± 4.4 years in controls, 8.1 ± 4.2 years in the CLD group, 4.5 ± 5.3 years in the ALF group, and 11.0 ± 5.3 years in the EHPVO group. One patient in the CLD group had undergone liver transplantation 14 months prior to enrollment. In the CLD group, 24 patients had chronic cholestasis. Splenomegaly was observed in 37 patients (33 CLD, 1 ALF, and 3 EHPVO), while portal hypertension was present in 32 patients (29 CLD and 3 EHPVO); of these, 15 patients (13 CLD and 2 EHPVO) had severe portal hypertension (Table 1).

Despite PT-INR prolongation, bleeding was uncommon: two patients experienced bleeding episodes (one in the CLD group and one in the EHPVO group), both due to esophageal variceal bleeding (Table 1). No thrombotic events were recorded in the cohort (Table 1). PT-INR was significantly higher in patients than in controls (*p* < 0.001), with the highest values observed in the ALF group. Fibrinogen, antithrombin (AT), protein C (PC), and several coagulation factors (FV, FVII, FIX, and FXI) were significantly lower in patients than in controls (all *p* < 0.001), whereas factor VIII (FVIII) was significantly higher (*p* < 0.001) (Table 2 and Table 3).

The biochemical parameters of patients and controls with liver disease are reported in Table 4. The data clearly demonstrate significant alterations in liver function markers across disease groups. Notably, AST and ALT are markedly elevated (*p* < 0.001), particularly in ALF. Similarly, total and direct bilirubin levels are increased. Conversely, albumin levels are reduced in patients, especially in CLD. Platelet counts are also decreased, most prominently in EHPVO (Table 4).

**Figure 1:**
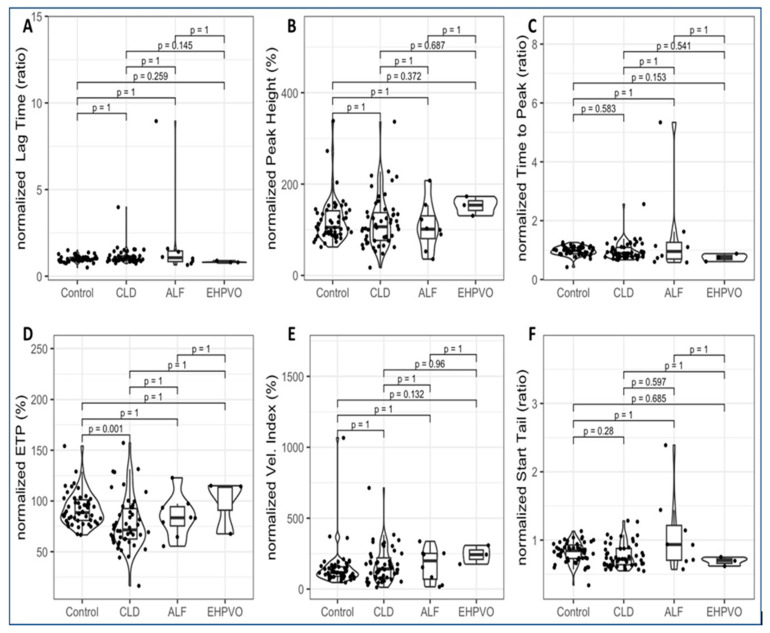
Comparison of thrombin generation parameters across study groups. Violin plots overlaid with boxplots and individual data points illustrate normalized thrombin generation parameters in controls, chronic liver disease (CLD), acute liver failure (ALF), and extrahepatic portal vein obstruction (EHPVO). Panels show (**A**) lag time ratio, (**B**) peak height ratio, (**C**) time-to-peak ratio, (**D**) endogenous thrombin potential (ETP) %, (**E**) velocity index %, and (**F**) start-tail ratio.

Thrombin generation was successfully performed in patients and controls. Group-wise results are summarized in Table 5 and illustrated in Figure 1.

Among thrombin generation parameters, only the normalized ETP showed a significant difference across groups, with reduced values observed in CLD compared with controls. In contrast, all other parameters, including lag time, peak height, time to peak, velocity index, and start tail, did not differ significantly (Figure 1).

Normalized lag time ratio was 1.0 ± 0.2 in controls and showed a trend toward higher values in ALF 2.0 ± 2.8 compared with CLD 1.1 ± 0.5 and EHPVO 0.8 ± 0.1 (Table 5; Figure 1A). Normalized peak height ratio was comparable between controls 120.7 ± 48.8, CLD 117.5 ± 57.3 and lower in ALF 108.2 ± 54.8, while higher values were observed in EHPVO 152.5 ± 21.2 (Table 5; Figure 1B). Normalized time-to-peak ratio was 1.0 ± 0.2 in controls, increased in ALF 1.5 ± 1.6, and lower in EHPVO 0.7 ± 0.1 (Table 5; Figure 1C).

Among thrombin generation metrics, normalized ETP ratio differed significantly across groups (*p* = 0.001), with lower values in CLD 78.0 ± 25.7 and ALF 84.9 ± 20.6 compared with controls 92.2 ± 16.2, while EHPVO showed values closer to controls 99.0 ± 27.2 (Table 5; Figure 1D). Normalized velocity index ratio and start tail ratio did not differ significantly across diagnostic groups (*p* = 0.192 and *p* = 0.068, respectively) (Table 5; Figure 1E,F).

**Figure 2:**
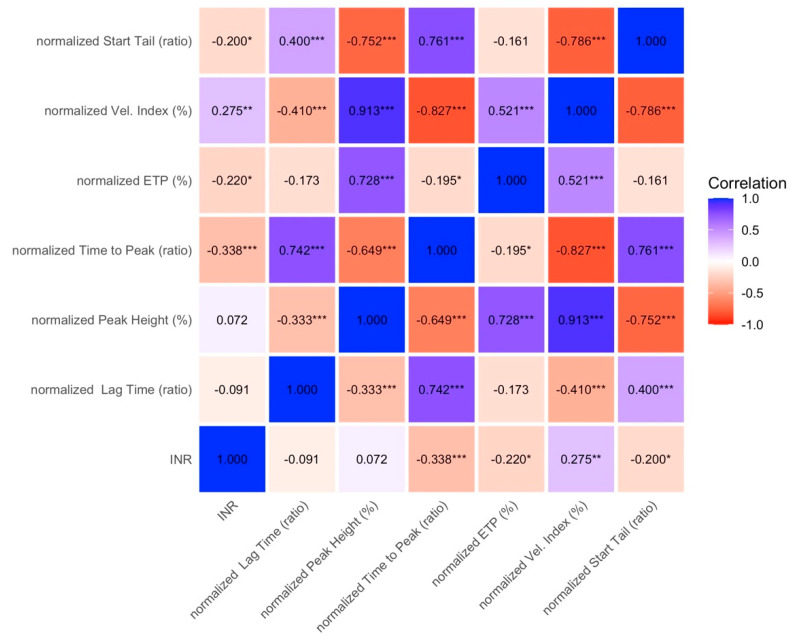
Spearman correlation matrix of thrombin generation parameters and INR. Colors represent the strength and direction of correlations, with blue indicating positive correlations and red indicating negative correlations. Values are expressed as correlation coefficients. Asterisks denote statistical significance (* *p* < 0.05, ** *p* < 0.01, *** *p* < 0.001). ETP: endogenous thrombin potential; INR: international normalized ratio.

Correlation analysis showed strong internal associations among thrombin generation parameters. In particular, normalized velocity index was strongly positively correlated with normalized peak height (r = 0.91, CI 95%: 0.86; 0.95) and strongly negatively correlated with normalized time to peak (r = −0.83, CI 95%: −0.88; −0.76) and start tail ratio (r = −0.79, CI 95%: −0.85; −0.69). Moderate correlations were observed between start tail ratio and time to peak (r = 0.76, CI 95%: 0.62; 0.85), between normalized ETP and peak height (r = 0.73, CI 95%: 0.62; 0.81), and between time to peak and lag time (r = 0.74, CI 95%: 0.60; 0.83); conversely, correlations between PT-INR and thrombin generation parameters were weak or absent (Figure 2).

**Figure 3:**
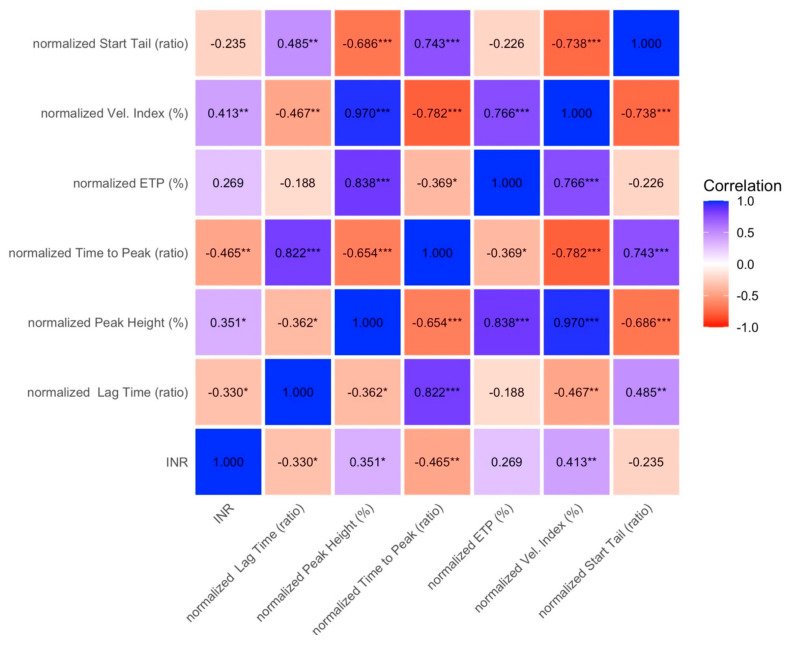
Spearman correlation between PT-INR and thrombin generation parameters in patients with lower disease severity, PELD/MELD s10, group A. Colors represent the strength and direction of correlations, with blue indicating positive correlations and red indicating negative correlations. Values are expressed as correlation coefficients. Asterisks denote statistical significance (* *p* < 0.05, ** *p* < 0.01, *** *p* < 0.001). ETP: endogenous thrombin potential; INR: international normalized ratio.

**Figure 4:**
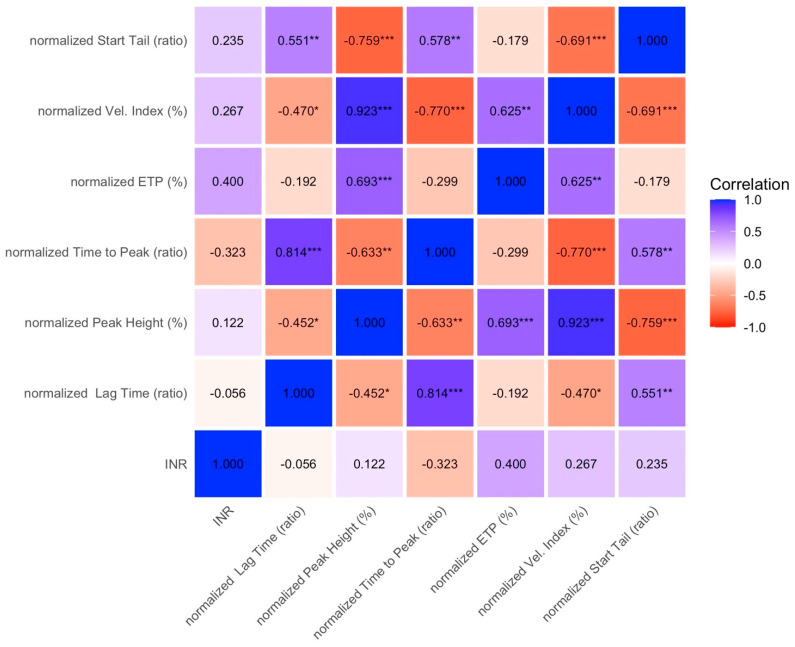
Spearman correlation matrix between PT-INR and thrombin generation parameters in patients with higher disease severity, PELD/MELD > 10, group B. Colors represent the strength and direction of correlations, with blue indicating positive correlations and red indicating negative correlations. Values are expressed as correlation coefficients. Asterisks denote statistical significance (* *p* < 0.05, ** *p* < 0.01, *** *p* < 0.001). ETP: endogenous thrombin potential; INR: international normalized ratio.

When patients were stratified by PELD/MELD score (Group A: ≤10; Group B: >10), PT-INR remained weakly or not correlated with thrombin generation indices in Group A (Figure 3), and no correlations were observed in Group B (Figure 4).

**Figure 5:**
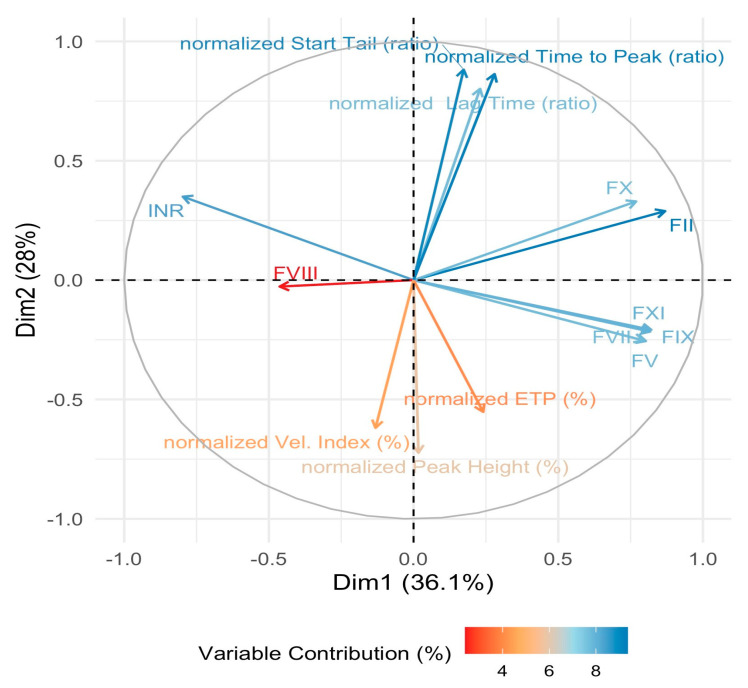
Principal component analysis (PCA) biplot showing variables and observations projected onto the first two principal components, which explain 36.1% and 28% of the total variance, respectively (cumulative variance: 64.1%). Variables are represented as vectors, with length and direction reflecting their contribution to the components.

Principal component analysis (PCA) was performed to explore the variance structure of thrombin generation parameters, coagulation factors, and PT-INR across the study population. The first two principal components accounted for 64.1% of the total variance. Dimension 1 was predominantly driven by thrombin generation parameters, which clustered together with high loadings. In contrast, dimension 2 was mainly influenced by INR and coagulation factors, indicating a variance structure distinct from thrombin generation indices. The PCA biplot showed a clear separation between thrombin generation parameters and conventional coagulation measures, supporting the concept that thrombin generation provides complementary information on the hemostatic profile of pediatric patients with liver disease. No clear clustering by diagnostic group was observed in the PCA space, suggesting that the main source of variance was related to the type of parameter rather than disease category (Figure 5).

**Figure 6:**
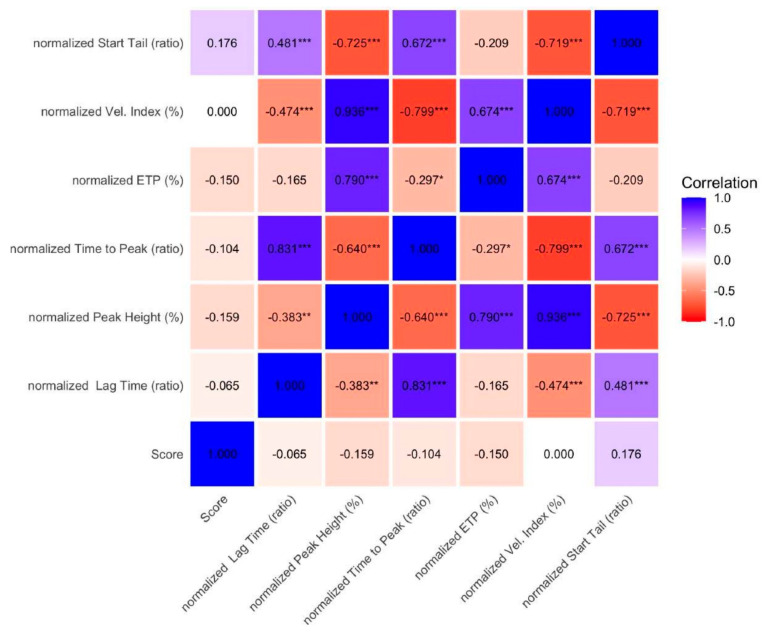
Spearman correlation matrix of PELD/MELD score and thrombin generation parameters. Values are expressed as correlation coefficients. Asterisks denote statistical significance (* *p* < 0.05, ** *p* < 0.01, *** *p* < 0.001).

The score was generated by PELD/MELD and no correlations were observed between score and thrombin generation parameters (Figure 6).

## 4. Discussion

Hemostasis is a multifaceted process involving tissue factor exposure, platelet activation, amplification through intrinsic and extrinsic pathways, and regulation by endogenous anticoagulants and fibrinolysis. An imbalance among these components ultimately determines whether the overall phenotype is thrombotic, balanced, or hemorrhagic [17]. Conventional coagulation test such as PT-INR primarily reflects the time to initial fibrin formation under standardized in vitro conditions and are particularly useful for monitoring vitamin K antagonist therapy. However, they provide limited information on anticoagulant pathways, cellular contributions (platelets and endothelium), and fibrinolytic activity, and therefore are not fully representative of in vivo hemostasis [18,19]. As a consequence, isolated PT-INR prolongation may be over-interpreted as “hypocoagulability,” potentially influencing transfusion strategies or procedural decision-making in ways that are not aligned with true bleeding risk.

Thrombin generation assay (TGA) has been proposed as global test that better capture the capacity to generate thrombin over time, thereby offering a more comprehensive view of hemostatic potential [20,21]. In adults with cirrhosis, normal thrombin generation has been described despite abnormal PT-INR, underscoring the limitations of conventional assays when the clinical question concerns global hemostasis [22]. TGAs quantify the kinetics and magnitude of thrombin formation, providing complementary information that may be relevant to both bleeding risk assessment and thrombotic tendency in selected settings [23].

Liver disease and coagulopathy are closely intertwined in children, in both chronic and acute settings. A clear understanding of the relationship between hepatic function and hemostasis is essential for clinical management, particularly because routinary coagulation tests are widely used to assess “coagulation status” and liver dysfunction, but clinical events do not necessarily mirror laboratory abnormalities [2]. In chronic liver disease, progressive fibrosis may evolve toward cirrhosis and advanced hepatic dysfunction, leading to complex alterations of both procoagulant and anticoagulant pathways. In addition, reduced portal flow velocity and endothelial injury may predispose to portal vein thrombosis [24].

Recent advances in non-invasive diagnostic approaches, including elastography and serum biomarkers, have significantly improved early detection of liver disease and reduced the reliance on invasive liver biopsy [25]. However, these tools primarily assess structural liver damage and do not provide information on functional aspects such as hemostatic balance.

However, the actual prevalence of thrombosis in pediatric liver disease varies across settings and populations. Conversely, clinicians are often primarily concerned about bleeding because of prolonged PT-INR; nevertheless, the reported incidence of major bleeding in pediatric chronic liver disease is relatively limited, generally ranging from 10% to 30%, and is predominantly driven by portal hypertension–related complications, most notably esophageal variceal bleeding [26,27].

From a clinical perspective, the potential utility of thrombin generation assay lies in their ability to improve decision-making in situations where conventional tests such as PT-INR are insufficient. The incorporation of thrombin generation assay could help identify patients with preserved global hemostatic capacity despite prolonged PT-INR, thereby avoiding unnecessary plasma transfusions or delays in invasive procedures such as liver biopsy. In addition, thrombin generation testing may be useful in selected high-risk scenarios, such as acute liver failure or advanced chronic liver disease, where both bleeding and thrombotic complications may occur. Overall, these findings suggest that thrombin generation assay may contribute to a more accurate and clinically meaningful assessment of coagulation status, moving beyond the limitations of PT-INR and supporting more tailored management strategies in pediatric liver disease.

Our findings show that PT-INR prolongation does not reliably reflect global hemostatic capacity in pediatric liver disease. Although PT-INR was significantly higher in patients than in controls, particularly in the ALF group, whereas bleeding events were uncommon. No thrombotic events were observed. This lack of correlation between abnormal conventional coagulation tests and clinical events supports the concept of rebalanced hemostasis, in which concomitant changes in procoagulant and anticoagulant pathways result in a compensated equilibrium. Patients showed reduced levels of fibrinogen, antithrombin, protein C anticoagulant, and several coagulation factors, together with increased factor VIII. This pattern suggests that liver disease does not simply lead to hypocoagulability, but rather alters both the procoagulant and anticoagulant components of the hemostatic system.

Among thrombin generation parameters, only normalized ETP showed a statistically significant difference between controls and CLD, whereas the other indices remained preserved. This suggests that, despite marked abnormalities in conventional coagulation and biochemical tests, thrombin generation kinetics remain preserved. Such preservation supports the view that the overall hemostatic profile in pediatric liver disease is more complex than suggested by PT-INR alone. Thrombin generation parameters showed strong internal associations, whereas correlations between PT-INR and TGA indices were weak or absent. Notably, this lack of correlation persisted even after stratification by PELD/MELD score, indicating that increasing disease severity does not improve the ability of PT-INR to reflect global hemostatic status.

In our study, the two girls who experienced bleeding belonged to the CLD and EHPVO groups, respectively, and had severe portal hypertension. Both showed a more “procoagulant” TGA profile compared with the median of their respective groups. INR was comparable to the group median in the first patient (1.40 vs. 1.41), whereas in the second patient INR was lower than the group median (1.45 vs. 1.62). The other coagulation and biochemical parameters were broadly comparable to the median values of their respective groups. Patients with CLD or EHPVO and severe portal hypertension, a bleeding event may depend more on portal hypertension than on a true inability of plasma to generate thrombin.

From a practical perspective, these findings suggest that TGA may be useful in clinical situations where PT-INR is abnormal but insufficient to define the actual coagulation profile.

This study has limitations, including its single-center design, the small sample size and the low number of clinical events in the ALF and EHPVO subgroups. Second, thrombomodulin-modified thrombin generation—which is particularly informative for assessing the protein C pathway and a prothrombotic tendency—could not be performed due to blood volume constraints in pediatric patients.

## 5. Conclusions

Our results indicate that PT-INR is an incomplete marker of coagulation status in pediatric liver disease, whereas thrombin generation testing provides additional and clinically relevant information on global hemostatic balance. These findings support the complementary role of TGA in the assessment of pediatric liver disease and justify further prospective studies aimed at defining its value in predicting bleeding and thrombotic outcomes.

## Figures and Tables

**Table 1:** Clinical characteristics of patients and controls. Data are expressed as number (percentage) for categorical variables and as mean (standard deviation, SD) or median (interquartile range, IQR) for continuous variables. CLD: chronic liver disease; ALF: acute liver failure; EHPVO: extrahepatic portal vein obstruction.

	Controls	CLD	ALF	EHPVO
	*N* = 51	*N* = 50	*N* = 8	*N* = 3
**Female gender, *n* (%)**	29 (56.9)	25 (50.0)	3 (37.5)	2 (66.7)
**Mean age, y (SD)**	11.1 (4.4)	8.1 (4.2)	4.5 (5.3)	11 (5.3)
**Median age, y (IQR)**	12 (7; 14)	8 (6; 11)	2 (1; 5)	13 (9; 14)
**Liver transplantation, *n* (%)**	0 (0.0)	1 (2.0)	0 (0.0)	0 (0.0)
**Chronic cholestasis, *n* (%)**	0 (0.0)	24 (48)	0 (0.0)	0 (0.0)
**Splenomegaly, *n* (%)**	0 (0.0)	33 (66)	1 (12.5)	3 (100.0)
**Portal hypertension, *n* (%)**	0 (0.0)	29 (58)	0 (0.0)	3 (100.0)
**Portal hypertension severe, *n* (%)**	0 (0.0)	13 (26)	0 (0.0)	2 (66.7)
**Bleeding, *n* (%)**	0 (0.0)	1 (2.0)	0 (0.0)	1 (33.3)
**Esophageal variceal, *n* (%)**	0 (0.0)	1 (2.0)	0 (0.0)	1 (33.3)
**Thrombosis, *n* (%)**	0 (0.0)	0 (0.0)	0 (0.0)	0 (0.0)

**Table 2:** Results of coagulation parameters in controls and patient groups. Data are expressed as mean (standard deviation, SD) and median (interquartile range, IQR). CLD: chronic liver disease; ALF: acute liver failure; EHPVO: extrahepatic portal vein obstruction. PT INR: prothrombin time international normalized ratio; aPTT: activated partial thromboplastin time; AT: antithrombin; D-D: D-Dimer; PC: Protein C anticoagulant; PS: Protein S free.

Parameter	Controls	CLD	ALF	EHPVO	*p* Value
	*N* = 51	*N* = 50	*N* = 8	*N* = 3	
**PT INR, mean (SD)**	1 (0.1)	1.4 (0.3)	2.1 (0.6)	1.7 (0.2)	
**PT INR, median (IQR)**	1.0 (0.9; 1.0)	1.4 (1.1; 1.6)	2 (1.9; 2.1)	1.7 (1.6; 1.8)	<0.001
**Fibrinogen (mg/dL), means (SD)**	298.4 (45.2)	275.8 (86)	154.1 (52.8)	182.7 (69.1)	
**Fibrinogen (mg/dL), median (IQR)**	295 (271; 334)	276 (197; 346)	157 (118; 193)	150 (143; 206)	<0.001
**aPTT sec, mean (SD)**	31.6 (2.8)	33.7 (5.5)	37.5 (8.7)	35.5 (4.6)	
**aPTT sec, median (IQR)**	31.6 (29.9; 33.8)	33.2 (30.1; 37.5)	35 (30.5; 42.2)	36.4 (33.5; 38)	0.060
**AT (%), mean (SD)**	103.5 (7.6)	93.1 (34.4)	54.2 (25.2)	85.3 (14.5)	
**AT (%), median (IQR)**	104 (99; 108)	97 (69; 107)	50 (37; 66)	78 (77; 90)	<0.001
**D-D (µg/mL) mean (SD)**	0.4 (0.3)	1.1 (1.8)	2.1 (2.7)	0.6 (0.5)	
**D-D (µg/mL) median (IQR)**	0.3 (0.3; 0.4)	0.3 (0.3; 0.9)	1.1 (0.6; 1.6)	0.6 (0.4; 0.8)	0.058
**PC (%) mean (SD)**	88.8 (15.3)	67.8 (35.1)	57.5 (69.3)	42 (-)	
**PC (%) median (IQR)**	88 (76; 100)	57 (37; 91)	35 (23; 37)	42 (42; 42)	<0.001
**PS (%) mean (SD)**	86 (13.3)	87.8 (34.9)	63.3 (33.9)	58 (-)	
**PS (%) median (IQR)**	86 (78; 93)	80 (69; 112)	54 (44; 61)	58 (58; 58)	0.054

**Table 3:** Results of factor levels in controls and patient groups. Data are presented as mean (standard deviation, SD) and median (interquartile range, IQR). CLD: chronic liver disease; ALF: acute liver failure; EHPVO: extrahepatic portal vein obstruction.

Parameter	Controls	CLD	ALF	EHPVO	*p* Value
	*N* = 51	*N* = 50	*N* = 8	*N* = 3	
**Factor II (%) mean (SD)**	100.2 (17.4)	74.4 (27.7)	74.2 (85.2)	-	
**Factor II (%) median (IQR)**	100 (89; 107)	75 (53; 89)	40 (30; 58)	-	<0.001
**Factor V (%) mean (SD)**	112.5 (22.5)	76.2 (37.4)	44.7 (21)	24 (-)	
**Factor V (%) median (IQR)**	110 (95; 126)	68 (51; 97)	39 (32; 48)	24 (24; 24)	<0.001
**Factor VII (%) mean (SD)**	98.9 (21.7)	74.6 (35.8)	20.8 (8.4)	-	
**Factor VII (%) median (IQR)**	99 (81; 110)	72 (47; 97)	22 (17; 26)	-	<0.001
**Factor VIII (%) mean (SD)**	115.5 (29.6)	189.8 (70.9)	243 (137.2)	-	
**Factor VIII (%) median (IQR)**	110 (98; 134)	173 (142; 237)	255 (138; 352)	-	<0.001
**Factor IX (%) mean (SD)**	87.6 (12.7)	70.6 (27.9)	55 (31.3)	-	
**Factor IX (%) median (IQR)**	88 (77; 98)	64 (50; 90)	55 (30; 85)	-	<0.001
**Factor X (%) mean (SD)**	94 (15.6)	79.2 (39.4)	77.8 (85.3)	-	
**Factor X (%) median (IQR)**	93 (82; 103)	75 (56; 86)	40 (39.2; 64.8)	-	<0.001
**Factor XI (%) mean (SD)**	113 (25.3)	73.2 (35.0)	48.3 (20.4)	-	
**Factor XI (%) median (IQR)**	110 (99.5; 117)	65 (48.5; 91)	39.5 (34.8; 63.8)	-	<0.001

**Table 4:** Biochemical parameters in controls and patients with liver disease. Results are reported as mean (standard deviation, SD) and median (interquartile range, IQR). Comparisons include patients with chronic liver disease (CLD), acute liver failure (ALF), and extrahepatic portal vein obstruction (EHPVO) versus controls. PLT: platelet count; Hb: hemoglobin; AST: aspartate aminotransferase; ALT: alanine ami-notransferase; γGT: gamma-glutamyl transferase.

Parameter	Controls	CLD	ALF	EHPVO	*p* Value
	*N* = 51	*N* = 50	*N* = 8	*N* = 3	
**PLT 10^3^/µL, (SD)**	252.2 (63.9)	152 (124.3)	221.1 (107.3)	74 (56.9)	
**PLT 10^3^/µL, (IQR)**	247 (217.5; 278.5)	123 (50.2; 245.2)	269 (126.8; 281.5)	50 (41.5; 94.5)	<0.001
**Hb g/dL (SD)**	13.4 (1.4)	11.7 (2)	10.6 (2)	11.2 (2.8)	
**Hb g/dL (IQR)**	13.1 (12.7; 13.9)	11.9 (10.3; 12.8)	9.6 (9.3; 12.3)	12.7 (10.3; 12.8)	<0.001
**AST U/L (SD)**	21.4 (7.5)	164.9 (252.6)	494.8 (369.6)	31 (9.2)	
**AST U/L (IQR)**	20 (15.5; 26)	58.5 (34; 189.2)	300.5 (249; 746.5)	29 (26; 35)	<0.001
**ALT U/L (SD)**	14.9 (6.4)	146.4 (204.2)	701.1 (861.8)	31 (9.2)	
**ALT U/L (IQR)**	14 (10; 17)	65.5 (25.2; 181)	471.5 (271.5; 628.5)	18 (16.5; 19)	<0.001
**γGT U/L (SD)**	11.1 (4)	105.8 (115.5)	152.3 (107.8)	18.3 (6.7)	
**γGT U/L (IQR)**	11 (9; 12)	59 (29; 118)	130 (88; 207.5)	20 (15.5; 22)	<0.001
**Bilirubin total mg/dL (SD)**	0.5 (0.2)	3.4 (5.1)	5.7 (4.5)	1.5 (1)	
**Bilirubin total mg/dL (IQR)**	0.5 (0.3; 0.6)	1.1 (0.6; 3.7)	5.5 (1.5; 8.8)	1.7 (1.1; 2.1)	<0.001
**Bilirubin direct mg/dL (SD)**	0.2 (0.2)	2.2 (3.7)	3.3 (2.6)	0.6 (80.4)	
**Bilirubin direct mg/dL (IQR)**	0.2 (0.1; 0.3)	0.5 (0.2; 2.3)	3.1 (1.0; 5.5)	0.6 (0.4; 0.8)	<0.001
**Albumin g/dL (SD)**	4.5 (0.4)	3.9 (0.6)	3.4 (0.5)	4.0 (0.6)	
**Albumin g/dL (IQR)**	4.5 (4.2; 4.8)	4.1 (3.5; 4.3)	3.5 (2.9; 3.7)	4.2 (3.8; 4.3)	<0.001

**Table 5:** Thrombin generation parameters comparison between controls and patients with liver disease. Normalized data are shown as mean (SD) and median (IQR). Patients with chronic liver disease (CLD), acute liver failure (ALF), and extrahepatic portal vein obstruction (EHPVO) are compared with healthy controls. Lag time, peak height, time to peak, endogenous thrombin potential (ETP), velocity index, and start tail are reported as ratios.

Parameter	Controls	CLD	ALF	EHPVO	*p* Value
	*N* = 51	*N* = 50	*N* = 8	*N* = 3	
**Lag time ratio mean (SD)**	1 (0.2)	1.1 (0.5)	2 (2.8)	0.8 (0.1)	0.120
**Lag time ratio median (IQR)**	1 (0.9; 1.1)	1 (0.9; 1.2)	1.1 (0.8; 1.5)	0.8 (0.8; 0.9)	
**Peak height ratio mean (SD)**	120.7 (48.8)	117.5 (57.3)	108.2 (54.8)	152.5 (21.2)	0.287
**Peak height ratio median (IQR)**	105.2 (90.4; 141.6)	106.6 (76.6; 137.4)	101.3 (80.6; 130.5)	153.9 (142.2; 163.4)	
**Time to peak ratio mean (SD)**	1 (0.2)	1 (0.3)	1.5 (1.6)	0.7 (0.1)	0.104
**Time to peak ratio median (IQR)**	1 (0.9; 1.1)	0.9 (0.8; 1.1)	1 (0.7; 1.3)	0.8 (0.7; 0.8)	
**ETP ratio mean (SD)**	92.2 (16.2)	78 (25.7)	84.9 (20.6)	99 (27.2)	0.001
**ETP ratio median (IQR)**	88.5 (80.8; 101.2)	71.5 (62.5; 92.5)	83.4 (75.4; 94.3)	114.3 (90.9; 114.7)	
**Velocity index ratio mean (SD)**	150.3 (146.5)	171.1 (124.5)	170.8 (118.7)	242.4 (66.4)	0.192
**Velocity index ratio median (IQR)**	116.2 (90.5; 157.1)	142.1 (77.9; 219.9)	199.2 (69.7; 251.6)	243.2 (209.4; 275.8)	
**Start tail ratio mean (SD)**	0.8 (0.2)	0.8 (0.2)	1.1 (0.6)	0.7 (0.1)	0.068
**Start tail ratio median (IQR)**	0.8 (0.7; 0.9)	0.7 (0.6; 0.9)	0.9 (0.7; 1.2)	0.7 (0.7; 0.7)	

## Data Availability

The data presented in this study are available from the corresponding author upon reasonable request. All data are anonymized and handled in accordance with institutional policies and applicable data protection regulations.

## Abbreviations

The following abbreviations are used in this manuscript:

PT-INR	Prothrombin time
TGA	Thrombin generation test
ALF	Acute liver failure
CLD	Chronic liver disease
PAI-1	Plasminogen activator inhibitor type 1
FII	Factor II
FV	Factor V
FVII	Factor VII
FX	Factor X
FVIII	Factor VIII
FIX	Factor IX
FXI	Factor XI
D-D	D-Dimer
AT	Antithrombin
PC	Protein C anticoagulant
EHPVO	Extrahepatic portal vein obstruction
ETP	Endogenous thrombin potential
PS	Protein S
